# Circadian Control of Dendrite Morphology in the Visual System of *Drosophila melanogaster*

**DOI:** 10.1371/journal.pone.0004290

**Published:** 2009-01-28

**Authors:** Paweł Weber, Elżbieta Kula-Eversole, Elżbieta Pyza

**Affiliations:** 1 Department of Cytology and Histology, Institute of Zoology, Jagiellonian University, Kraków, Poland; 2 Department of Biology, Howard Hughes Medical Institute and National Center for Behavioral Genomics, Brandeis University, Waltham, Massachusetts, United States of America; Yale School of Medicine, United States of America

## Abstract

**Background:**

In the first optic neuropil (lamina) of the fly's visual system, monopolar cells L1 and L2 and glia show circadian rhythms in morphological plasticity. They change their size and shape during the day and night. The most pronounced changes have been detected in circadian size of the L2 axons. Looking for a functional significance of the circadian plasticity observed in axons, we examined the morphological plasticity of the L2 dendrites. They extend from axons and harbor postsynaptic sites of tetrad synaptic contacts from the photoreceptor terminals.

**Methodology/Principal Findings:**

The plasticity of L2 dendrites was evaluated by measuring an outline of the L2 dendritic trees. These were from confocal images of cross sections of L2 cells labeled with GFP. They were in wild-type and clock mutant flies held under different light conditions and sacrified at different time points. We found that the L2 dendrites are longest at the beginning of the day in both males and females. This rhythm observed under a day/night regime (LD) was maintained in constant darkness (DD) but not in continuous light (LL). This rhythm was not present in the arrhythmic *per^01^* mutant in LD or in DD. In the clock photoreceptor *cry^b^* mutant the rhythm was maintained but its pattern was different than that observed in wild-type flies.

**Conclusions/Significance:**

The results obtained showed that the L2 dendrites exhibit circadian structural plasticity. Their morphology is controlled by the *per* gene-dependent circadian clock. The L2 dendrites are longest at the beginning of the day when the daytime tetrad presynaptic sites are most numerous and L2 axons are swollen. The presence of the rhythm, but with a different pattern in *cry^b^* mutants in LD and DD indicates a new role of *cry* in the visual system. The new role is in maintaining the circadian pattern of changes of the L2 dendrite length and shape.

## Introduction

The visual system of different fly species show circadian rhythms in many physiological and structural processes. They have been detected in the retina, in changes of the electroretinogram amplitude [1], and in the first optic neuropil (lamina). In the lamina they include rhythms in the number of presynaptic profiles [2], in changes of interneuron axon sizes [3], [4] and in migration of screening pigment granules in the photoreceptor terminals [5]. Those circadian rhythms existing in the retina photoreceptors are probably generated by circadian oscillators, called peripheral oscillators, located in the photoreceptors themselves [6]. The photoreceptors, like the clock neurons in the brain, the so-called lateral (LNs) and dorsal (DNs) neurons, show expression of clock genes. In the clock neurons the main clock gene *period* (*per*) and its protein (PER), oscillate in a circadian pattern [7]–[10]. In contrast to the retina photoreceptors, an expression of clock genes has not been observed in the lamina neurons that show circadian structural plasticity. PER staining, however, has been detected in the lamina glial cells [10]. In the retinal photoreceptors in *D. melanogaster* PER staining is the highest at the end of the night and begins to decrease at the beginning of the day [9]. While intensity of PER staining during the night is the same in both the LNs and the photoreceptors, during the day it decays faster in photoreceptors and glia than in the LNs [9]. The molecular clock in most clock cells is based on circadian transcription and translation feedback loops with two main core genes, *per* and *timeless* (*tim*). It is entrained by light and other environmental cues to drive daily rhythms in output processes [11]. The circadian clock in the LNs is entrained by light inputs mainly from the extraretinal photoreceptors of the Hofbauer-Buchner eyelet. It is entrained by light inputs also from the compound eyes. In addition it possesses its own photoreceptor cryptochrome (CRY), the blue-light absorbing protein, encoded by the *cryptochrome* (*cry*) gene [12]. CRY is responsible for light-dependent degradation of *tim* protein TIM and resetting the circadian rhythms. In turn TIM is required for stabilization of PER. CRY might also be an element of the molecular circadian clock, as it has been suggested in peripheral oscillators [13], [14]. In the current model of the molecular clock in the pacemaker neurons in the brain of *Drosophila*, the transcription factors CLOCK (CLK) and CYCLE (CYC) activate expression of *per* and *tim*, and PER and TIM accumulate and form PER/TIM heterodimers in the cytoplasm in darkness. About 6 h after lights-off they enter the nucleus, dissociate and PER represses transcription by preventing the CLK/CYC heterodimers from binding to *per* and *tim* promotores [15]. The timing of PER, TIM and other clock protein functions, their stability and subcellular localization depend on phosphorylation [16], [17]. In the nucleus PER and TIM do not accumulate simultaneously in time suggesting that those proteins do not act only as heterodimers and have distinct functions in the nucleus [18].

The rhythms detected in the lamina of the fly's optic lobe seem to be controlled by a circadian clock located in the brain [19]. An involvement of circadian oscillators in the photoreceptors and in the optic lobe glia is possible, however. The oscillation in size of two lamina large monopolar cells L1 and L2 have been detected in three flies species; *Musca domestica*, *Drosophila melanogaster* and *Calliphora vicina* [3], [4], [20]. In the housefly the axons of L1 and L2 cells change their girth during the day and night. This rhythm is maintained in constant darkness (DD) and in continuous light (LL). They are endogenously generated by a circadian clock. The daily pattern of these changes is species-specific and correlated with the locomotor activity pattern. Both cells are larger when locomotor activity of a fly increases [21]. In *D. melanogaster* L1 and L2 cells swell at the beginning of both the day and night. In the case of L2, in addition to axons, circadian oscillations have also been detected in size of nuclei but not in cell bodies [22]. As it has been found in *M. domestica*, structural changes in L1 and L2 monopolar cells are offset by changes in the lamina epithelial glial cells [23].

The function of rhythmic remodeling of L1 and L2 axons is still unknown. The aim of the present study was to examine the L2 dendrite morphology which may affect the size of the L2 axon. The dendrites extend from the L2 axons and surround the photoreceptor terminals in the lamina. They receive photic and visual information as postsynaptic sites through tetrad synapses from the photoreceptors. We hypothesized that changes in the size of L1 and L2 axons are correlated with changes in the size of dendrites. The length of the dendrites, in turn, is correlated with the number of tetrad synapses. In the housefly it has been found that tetrad presynaptic structures, called T-bars, show daily changes in frequency. The highest number of tetrad presynaptic profiles has been found when axons of both cells L1 and L2 were the largest [2].

**Figure 1:**
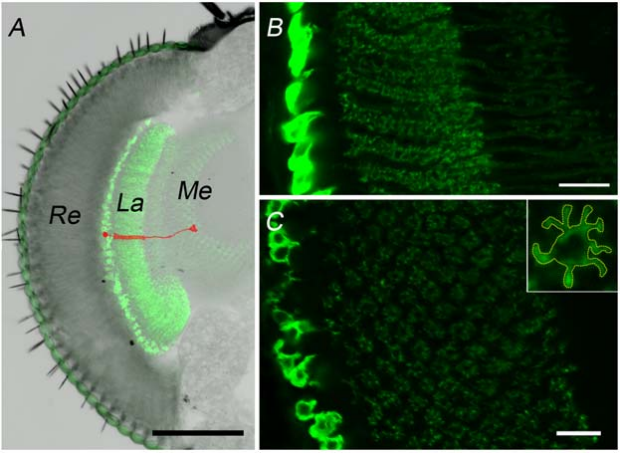
Morphology of L2 monopolar cells in the first optic neuropil (lamina) of *Drosophila melanogaster*. A. The optic lobe of *D. melanogaster* transgenic line (21D-GAL4×UAS-GFP-mCD8) with targeted expression of GFP to cell membrane of the L2 cells. The L2 cell bodies are located in the proximal lamina (lamina cortex), axons with dendrites in the lamina synaptic neuropil and terminals of axons in the second optic neuropil (medulla) Details of morphology of one L2 cell is drawn in red. B. Longitudinal section of the lamina with GFP-labeled L2 cells. C. Cross section of the lamina with GFP-labeled L2 cells. Inset: Cross section of a single L2 cell that was used for tracing the L2 dendritic tree. The outline of dendritic tree is marked with dashed line. Re – retina, La – lamina, Me – medulla. Scale bars: 100 µm (A), 10 µm (B,C).

**Figure 2:**
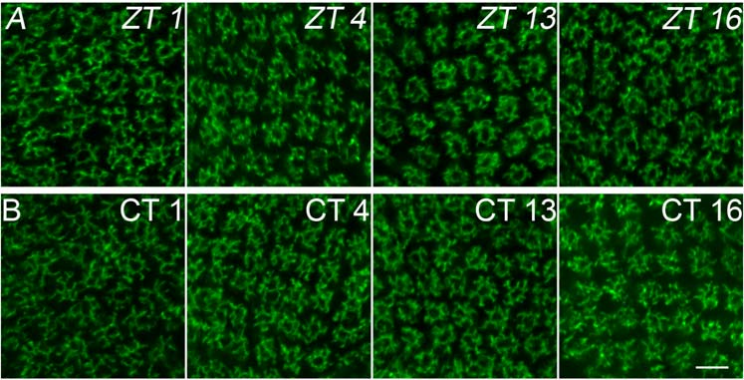
Confocal images of cross sections of the L2 dendritic trees. A. Fruit flies were sacrified at different times: ZT1, ZT4, ZT13, ZT16 (ZT0 – the beginning of the day, ZT12 – the beginning of the night) in the day/night LD 12∶12 condition. B. Fruit flies were sacrified at different times: CT1, CT4, CT13, CT16 (CT0 – the beginning of the subjective day, CT12 – the beginning of the subjective night) in constant darkness (DD). Scale bar: 5 µm.

**Figure 3:**
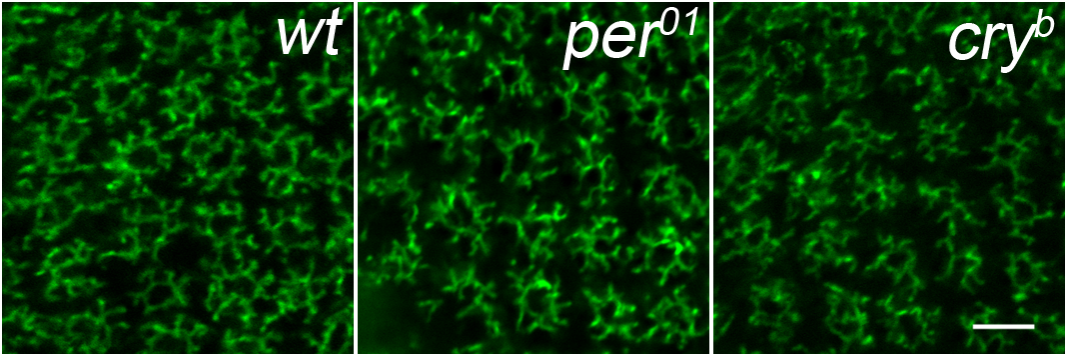
Confocal images of cross section of the L2 dendritic tree in different *D. melanogaster* strains. The L2 dendritic trees in: wt -21D-GAL4×UAS-mCD8-GFP; *per^01^* - *per^01^*;21D/TM2 GAL4×UAS-mCD8-GFP; *cry^b^* - *cry^b^*21D/TM6B GAL4×UAS-mCD8-GFP held in the day/night LD 12∶12 condition and sacrified at ZT1 – 1 h after lights-on.

Here we examined the length of the dendrites of the L2 monopolar cell. We did this by measuring the cross-sectional outline (size) of the L2 dendritic tree under different light conditions and at different times during the 24 h period. It was done on transgenic lines with targeted expression of green fluorescence protein (GFP) in the L2 cells in wild-type background *D. melanogaster* (Fig. 1,2) and in the arrhythmic *per^01^* null mutant and *cry^b^* mutant with an amino-acid substitution in CRY (Fig. 3). We found that the size of L2 dendritic tree changes during the day and night and that this structural plasticity is controlled by a circadian clock. Using *cry^b^* mutants we also detected that CRY is not only the circadian photoreceptor. It also controls the phase of the circadian rhythm in changes of the L2 dendrite morphology.

## Results

### Morphological plasticity of the L2 dendrites in wild-type *Drosophila*

#### LD12∶12

The daily statistically significant differences in the cross–sectional size of the L2 dendritic tree were found in both males and females (p<0.05) (Fig. 2A). There were no statistically significant differences in the L2 dendritic tree size between males and females, however. The morphology of the L2 dendritic tree was also similar in males and females, usually we observed 8 dendritic spines per cell (Fig. 3).

**Figure 4:**
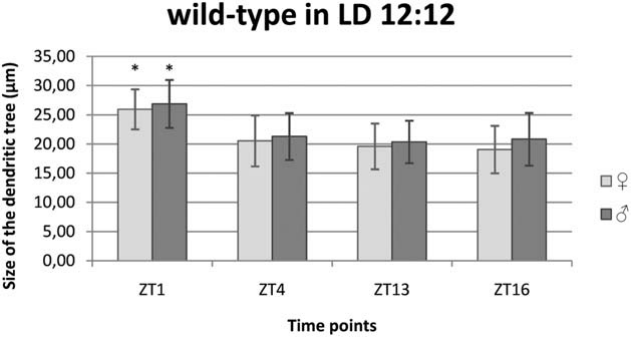
Daily changes of the size of the L2 dendritic tree in LD 12∶12. Flies of 21D-GAL4×UAS-mCD8-GFP *D. melanogaster* line were held in the day/night (LD 12∶12) condition and sacrificed at ZT1 – 1 h after lights-on, ZT4 – 4 h after lights-on, ZT13 – 1 h after lights-off, ZT16 – 4 h after lights-off. In LD 12∶12 the L2 dendritic tree was the largest at the beginning of the day in both males and females.

In females the size of the dendritic tree was the largest at ZT1 (25.95 µm±3.43 SD) and the smallest in the middle of the night (ZT16: 19.05 µm±4.05 SD) (Fig. 4). This means it was smaller by 27%. This difference was statistically significant (F = 41.33; p<0.001). At another time point studied (ZT4: 20.53 µm±4.35 SD and ZT13: 19.59 µm±3.92 SD) the L2 dendritic tree size was not statistically different from that at ZT1.

Similarly to females, the cross–sectional size of the L2 dendritic tree in males was the largest at ZT1 (26.86 µm±4.10 SD) but the smallest at the beginning of the night – ZT13 (20.36 µm±3.63 SD) (Fig. 4). When compared with ZT1, the L2 dendritic tree size was smaller by 21% at ZT4 (21.29 µm±4.02 SD), by 24% at ZT13 (20.36 µm±3.63 SD) and by 22% at ZT16. These differences were statistically significant (F = 28.46; p<0.001). No significant differences were detected between ZT4, ZT13 and ZT16.

In addition to changes in the size of the dendritic tree, there were also changes in morphology of the L2 dendritic spines which were long and thin during the day but short and thick during the night (Fig. 2A). These changes were observed in both sexes.

#### DD condition

**Figure 5:**
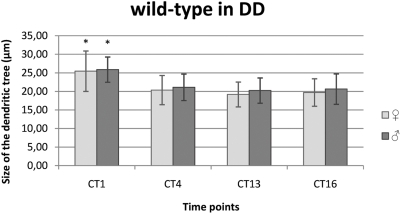
Circadian changes of the size of the L2 dendritic tree in DD. Flies of 21D-GAL4×UAS-mCD8-GFP *D. melanogaster* line were held in constant darkness (DD) and sacrificed at CT1 – 1 h from the beginning of subjective day, CT4 – 4 h from the beginning of the subjective day, CT13 – 1 h from the beginning of subjective night, CT16 – 4 h from the beginning of subjective night. In DD the L2 dendritic tree was the largest at the beginning of subjective day in both males and females.

In DD the pattern of size changes of the L2 dendritic tree was similar to that one detected in LD 12∶12 (Figs 2B,5). This indicated a circadian rhythm in changes of morphology of the L2 dendrites. No significant differences were found between males and females.

In females the size of the dendritic tree was the largest at CT1 (25.46 µm±5.45 SD) and smaller by 20% (CT4: 20.36 µm±3.92 SD), 25% (CT13: 19.18 µm±3.34 SD) and 23% (CT16: 19.71 µm±3.70 SD) at other time points (Fig. 5). These differences were statistically significant (F = 16.44; p<0.001). No significant differences were found between CT4, CT13 and CT16.

In males, the cross–sectional size of the L2 dendritic tree was the largest at CT1 (25.87 µm±3.40 SD) and the smallest at the beginning of the subjective night – CT13 (20.23 µm±3.39 SD). This difference was statistically significant (F = 41.61; p<0.001) (Fig. 5). The size was smaller at CT4 (21.11 µm±3.57 SD), CT13 and CT16 (20.64 µm±4.10 SD) than at CT1 by 18%, 22% and 20%, respectively. No statistically significant differences were detected between other time points, CT4, CT13 and CT16.

#### LL condition

**Figure 6:**
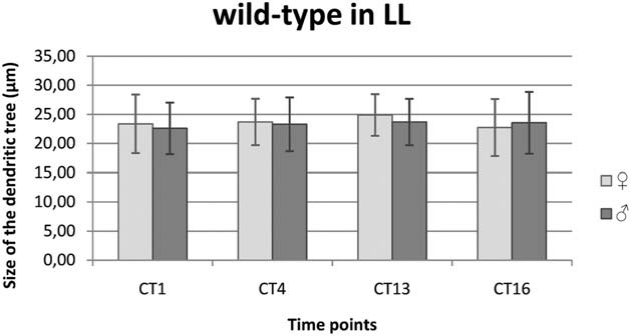
The size of the L2 dendritic tree in LL. Flies of 21D-GAL4×UAS-mCD8-GFP *D. melanogaster* line were held in continuous light (LL) and sacrificed at CT1 – 1 h from the beginning of subjective day, CT4 – 4 h from the beginning of the subjective day, CT13 – 1 h from the beginning of subjective night, CT16 – 4 h from the beginning of subjective night. There was not significant changes in the size of L2 dendritic tree during the subjective day and subjective night in males and females.

There was not significant changes in the size of the L2 dendritic tree during the subjective day and night in LL in males and females as well as between these groups (Fig. 6).

### Morphology of the L2 dendrites in *Drosophila* arrhythmic mutant *per^01^*

#### LD 12∶12

**Figure 7:**
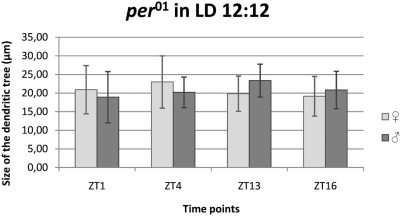
The size of the L2 dendritic tree in *per^01^* arrhythmic mutant in LD 12∶12. Flies of 21D-GAL4×UAS-mCD8-GFP in *per^01^* background *D. melanogaster* line were held in the day/night LD 12∶12 condition and sacrificed at ZT1 – 1 h after lights-on, ZT4 – 4 h after lights-on, ZT13 – 1 h after lights-off, ZT16 – 4 h after lights-off. There was not statistically significant differences in the L2 dendritic tree in both males and females at different times of the day and night.

There were not statistically significant differences in the cross–sectional sizes of the L2 dendritic tree during the day and night in males or in females (Fig. 7).

In addition to disruption of the circadian rhythm in the L2 dendrite plasticity in *per^01^* mutants, there were also observed changes in morphology of dendritic spines that were shorter in *per^01^* mutants than in wild-type flies at ZT1 (Fig. 3).

#### DD condition

**Figure 8:**
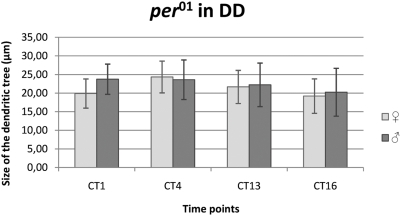
The size of the L2 dendritic tree in *per^01^* arrhythmic mutant in DD. Flies of 21D-GAL4×UAS-mCD8-GFP in *per^01^* background *D. melanogaster* line were held in constant darkness (DD) and sacrificed at CT1 – 1 h from the beginning of subjective day, CT4 – 4 h from the beginning of the subjective day, CT13 – 1 h from the beginning of subjective night, CT16 – 4 h from the beginning of subjective night. There was not significant differences in the size of L2 dendritic tree during the subjective day and subjective night in males and in females.

No significant differences were found in the cross–sectional sizes of the L2 dendritic tree in *per^01^* females or males held in DD (Fig. 8).

#### LL condition

**Figure 9:**
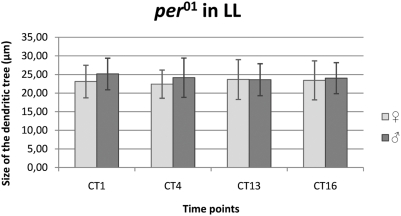
The size of the L2 dendritic tree in *per^01^* arrhythmic mutant LL. Flies of 21D-GAL4×UAS-mCD8-GFP in *per^01^* background *D. melanogaster* line were held in continuous light (LL) and sacrificed at CT1 – 1 h from the beginning of subjective day, CT4 – 4 h from the beginning of the subjective day, CT13 – 1 h from the beginning of subjective night, CT16 – 4 h from the beginning of subjective night. In LL significant differences in the size of L2 dendritic tree were not detected during the subjective day and subjective night.

There were no statistically significant differences in the cross–sectional sizes of the L2 dendritic tree in *per^01^* males or females held in LL (Fig. 9).

### Morphology of the L2 dendrites in *Drosophila* clock photoreception mutant *cry^b^*

#### LD 12∶12

**Figure 10:**
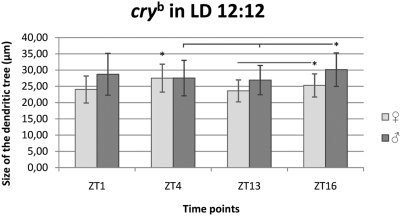
Changes of the size of the L2 dendritic tree in *cry^b^* mutant in LD 12∶12. Flies of 21D-GAL4×UAS-mCD8-GFP in *cry^b^* background *D. melanogaster* line were held in the day/night LD 12∶12 condition and sacrificed at ZT1 – 1 h after lights-on, ZT4 – 4 h after lights-on, ZT13 – 1 h after lights-off, ZT16 – 4 h after lights-off. The daily differences in the size of the L2 dendritic tree were observed in males and females, however, the pattern of changes was different than in wild-type background 21D-GAL4×UAS-GFP-mCD8 line.

In *cry^b^* flies the pattern of the L2 dendritic tree size changes was different in males than in females (Fig. 10) and dendritic spines were less regular in shape than in wild-types flies (Fig. 3). Some dendritic spines of one cell we longer than others in *cry^b^* while in wild-type flies all spines had mostly similar size.

In females the largest size was at ZT4 (27.53 µm±4.30 SD) and smaller at other time points by 13%, 14% and 8% at ZT1 (24.04 µm±4.17 SD, p<0.001), ZT13 (23.61 µm±3.38 SD, p<0.001) and ZT16 (25.30 µm±3.56 SD, p = 0.0036), respectively. These differences were statistically significant (F = 15.28; p<0.001). A significant difference was also between ZT13 and ZT16 (p = 0.034) but not between other time points.

In males the largest size of the dendritic tree was at ZT16 (30.15 µm±5.17 SD) and 9% smaller at ZT4 (27.55 µm±5.45 SD, p = 0.022) and 11% smaller at ZT13 (26.94 µm±4.50 SD, p = 0.002). These differences were statistically significant (F = 5.097; p<0.002). The differences were not statistically significant between ZT4 and ZT13, and ZT1 (28.72 µm±6.44 SD) and at other time points.

#### DD condition

**Figure 11:**
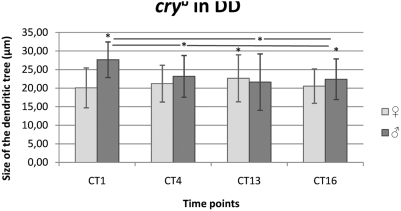
Changes of the size of the L2 dendritic tree in *cry^b^* mutant in DD. Flies of 21D-GAL4×UAS-mCD8-GFP in *cry^b^* background *D. melanogaster* line were held in constant darkness (DD) and sacrificed at CT1 – 1 h from the beginning of subjective day, CT4 – 4 h from the beginning of the subjective day, CT13 – 1 h from the beginning of subjective night, CT16 – 4 h from the beginning of subjective night. The size of L2 dendritic tree was slightly different at different time points in females but showed large differences in males. The pattern of changes was similar neither to that detected in wild-type background 21D-GAL4×UAS-mCD8-GFP flies in DD nor to that of *cry^b^* mutants in LD 12∶12.

Similarly to the LD condition, in DD, males had a different pattern of morphological changes of dendrites than females (Fig. 11). The sizes of the L2 dendritic tree in females were similar at all times examined, however, statistically significant differences were detected (F = 5.822; p<0.001). They were between CT13 (22.64 µm±6.32 SD) and other time points: CT1 (11%, 20.07 µm±5.36 SD, p = 0.02), CT4 (6%, 21.22 µm±4.96 SD, p<0,001) and CT16 (9%, 20.54 µm±4.62 SD, p = 0.01) but not between CT1, CT4 and CT16.

There were also statistically significant differences in the size of the dendritic tree in males during the subjective day and subjective night in DD (F = 196.41; p<0.001). The size was the largest at CT1 (27.65 µm±4.79 SD) and smaller by 16%, 22% and 19% at CT4 (23.17 µm±5.62 SD, p<0.001), CT13 (21.61 µm±7.59 SD, p<0.001), CT16 (22.39 µm±5.48 SD, p<0.001), respectively.

#### LL condition

**Figure 12:**
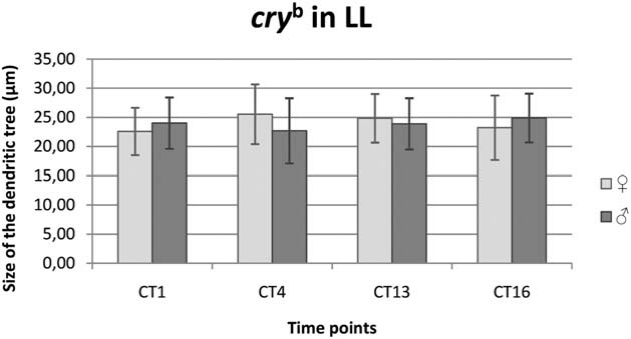
The size of the L2 dendritic tree in *cry^b^* mutant in LL. Flies of 21D/GAL4×UAS-mCD8-GFP in *cry^b^* background *D. melanogaster* line were held in continuous light (LL) and sacrificed at CT1 – 1 h from the beginning of subjective day, CT4 – 4 h from the beginning of the subjective day, CT13 – 1 h from the beginning of subjective night, CT16 – 4 h from the beginning of subjective night. Neither males nor females showed statistically significant changes in the size of L2 dendritic tree during the subjective day and subjective night.

In females there were no statistical significant differences in the size of the L2 dendritic tree between all time points (CT1- 22.57 µm±4.06 SD, CT4 – 25.52 µm±5.13 SD, CT13 – 24.84 µm±4.18 SD and CT16 – 23.22 µm±5.53 SD) (Fig. 12). In males, no significant differences were detected between all time points examined (CT1- 24.00 µm±4.42 SD, CT4 – 22.69 µm±5.60 SD, CT13 – 23.88 µm±4.40 SD and CT16 – 24.87 µm±4.19 SD) (F = 2.02, p>0.05) (Fig. 12).

## Discussion

### Circadian rhythm in morphology of dendrites

The results obtained in the present study showed that the L2 cell dendrites, postsynaptic sites of tetrad synapses in the first optic neuropil of *D. melanogaster*, show circadian plasticity in morphology. Under the day/night regime LD 12∶12, the L2 dendrites, measured as the cross-sectional outline of the dendritic tree are the longest at the beginning of day and shorter later during the day and at night. This daily rhythm in changes of size of the dendritic tree was maintained in DD. This indicates that the circadian plasticity of the L2 dendrites is controlled endogenously by a circadian clock. This rhythm was detected in both males and females. It had the similar pattern in both sexes under both conditions. The rhythm was not observed, however, in LL. Similarly to circadian rhythms in behavior, continuous light abolished the circadian plasticity of dendrites. It confirms other studies that the molecular mechanism of the clock in *D. melanogaster* is disrrupted in LL and PER protein is absent in the retina photoreceptors and in the LNs [9]. In contrast to the present observation, the results obtained in our earlier studies showed that daily oscillations in L1 and L2 axon sizes of the housefly are maintained in both DD and LL. Moreover, L1 and L2 mean sizes are larger in LL than in LD 12∶12 and are smallest in DD [3]. The differences in the size of L2 dendritic tree in LL and under different light conditions were not observed in *D. melanogaster*.

In LD 12∶12, L1 and L2 cells of *M. domestica* increase their axons during the day and show a decrease during the night. In turn, our study of L1 and L2 interneuron plasticity of *D. melanogaster* at electron microscope level has shown that both monopolar cells change their cross-sectional area of axons twice during the 24 h period. This change takes place at the beginning of the day and at the beginning of the night [4]. The observed differences between *M. domestica* and *D. melanogaster* indicate species specificity of visual and circadian systems. Circadian plasticity of L1 and L2 interneurons, however, have been found in both species. Moreover, in both species we have also found a correlation between the size of L1 and L2 interneurons and the level of locomotor activity [3], [4], [21]. Both cells swell when flies are highly active. The pattern of circadian L1 and L2 plasticity is synchronized with their species-specific pattern of the circadian rhythm in locomotor activity. In the present study we did not observe two peaks in the size of the L2 dendritic tree during the 24 h period but only one at the beginning of the day. It means that the largest L2 dendritic tree forms when the girth of the L2 axon increases at the beginning of the day. The second peak in changes of L1 and L2 axon size may be connected to other processes going on in monopolar cells at the beginning of the night or indicate that changes of axon sizes are not the same along the axons. In *D. melanogaster* the L2 axons change shape from an inverted conical form during the day to a cylindrical one at night [4]. The previous measurements of L1 and L2 axons were carried out at the proximal depth of the lamina while the present ones at more distal depth. In addition to axons, the L2 nuclei and cell bodies have also been studied in *Drosophila* transgenic line with GFP targeted to the L2 cytoplasm. This study showed that the nuclei are also larger during the day than during the night. In contrast, L2 cell bodies do not change their sizes during the day and night [22].

### The role of *per* and *cry* genes in control of circadian plasticity and morphology of the L2 dendrites

Using the transgenic flies in *per^01^* background we found that the observed circadian changes in size of the L2 cell dendritic tree were abolished in the null *per^01^* mutant flies. It confirms that the circadian plasticity of the L2 dendrites is controlled by the circadian clock for which *per* is crucial in its molecular mechanism. Unlike to the circadian rhythm in locomotor activity, the rhythm in the L2 dendrite plasticity in the *per^01^* mutants was not entrained by external daily changes of light and darkness. It means that the circadian input is necessary for maintaining circadian plasticity of dendrites also in LD conditions. The rhythm in circadian plasticity of L1 and L2 axons have also not been observed in the *per^01^* mutant in LD 12∶12 (Pyza E. and Meinertzhagen I.A., unpublished results).

In the present study we also examined involvement of *cry* gene in the circadian plasticity of dendrites. Cryptochrome has been suggested as an element of molecular mechanism of the circadian clock in peripheral clocks [13], [14], [25], [26]. This is in addition to its role as a circadian photoreceptor in the central pacemaker neurons [27], [28]. Since CRY has been detected in photoreceptors of the compound eyes [25], [26], CRY may play both functions in these peripheral oscillators. In the lamina, however, circadian rhythms may be regulated by the central pacemaker, at least in the housefly. This is because the severance of the optic lobe in this species abolishes the rhythm in swelling and shrinking of L1 and L2 axons [19]. In *D. melanogaster*, however, we have found that *per* expression in the LNs is not sufficient to generate the circadian plasticity of L1 and L2 monopolar cells (Pyza E, Hardin PE, Meinertzhagen IA, unpublished results). This rhythm was also not completely rescued after *per* expression in both the LNs and the retina photoreceptors. It indicates that *per* expression in additional cells, probably glial ones is also important for this rhythm. In case of dendrites their rhythmic morphological changes are correlated with circadian rhythms in the photoreceptor terminals, in the abundance of a presynaptic active zone protein Bruchpilot (Górska-Andrzejak J, Niańko E, Pyza E, unpublished results).

In the housefly, the pacemaker neurons transmitting circadian information to the lamina are probably the large PDF-immunoreactive neurons in the accessory medulla, similar to *Drosophila*'s large ventral LNs. Their terminals in the lamina and medulla show circadian structural changes [4] and cyclically release PDF [29] that affects target neurons and/or glial cells. In *D. melanogaster* circadian information from the central pacemaker neurons to the visual system are probably transmitted to *per*-expressing glial cell in the medulla. They may intermediate between the central clock and target cells in the visual system [30]. In addition circadian rhythms in the lamina may be controlled by circadian oscillators in glia and in the retina photoreceptors.

The results obtained in the present study indicate that CRY is important for setting the phase of the circadian rhythm in changes of the L2 dendrite morphology. In both LD and DD conditions in the transgenic flies in *cry^b^* background, the peak in size of the dendritic tree was shifted and the daily pattern of dendrite size changes was different than in wild-type flies.

In addition to the involvement of *per* and *cry* genes in regulating circadian plasticity of the L2 interneuron structure, mutations of both genes also affect morphology of the L2 dendrites. It means that both genes may play several, at least two, roles in the nervous system. They maintain structure and cyclical structural changes of neurons in the fly's visual system. This role they may also play in other parts of the nervous system [31].

### Function of circadian plasticity of dendrites

The circadian rhythms in the lamina are probably generated by several circadian oscillators. They receive circadian inputs originating from the central pacemaker neurons and clock gene expressing cells in the visual system. If they receive the circadian input from clock neurons in the central pacemaker, these neurons are different from those generating circadian rhythms in behavior. This is because they are not driven in *per^01^* mutants by light. It is also because *cry* is not only the circadian photoreceptor but also involved in the clockwork. Since the number of the tetrad presynaptic T-bars changes during the day and night, dendritic spines of the L2 interneurons, postsynaptic sites of these synaptic contacts, have to enlarge in synchrony. They have to enlarge in synchrony to the presynaptic active zone changes in the presynaptic cells. Later during the day and in the night they shorten. A space inside the cartridge is filled with glial cells which during the day decrease their sizes [23]. Because the optic lobes intermediate between the compound eyes and the central brain, rhythmic processes in the optic lobes are probably affected by circadian oscillators located in the brain and in the compound eye photoreceptors. In addition, they also synchronize rhythmic events occurring in both sites. Rhythmic remodeling of postsynaptic neurons controlled by circadian oscillators in the brain and in the visual system itself, may provide efficient transmission of photic and visual information. This is information which takes place during high behavioral activity of an animal.

## Materials and Methods

We used *Drosophila melanogaster* strains with the bipartite GAL4/UAS expression system [24] and generated flies that express GFP in the lamina L2 monopolar cells (Fig. 1). GAL4 enhancer trap line 21D-GAL4 which shows expression of GAL4 in L2 cells was kindly provided by Dr. Thomas Raabe (Würzburg Univ., Germany). The 21D-GAL4 strains with null mutation of gene *period* (*per ^01^*;21D/TM2) or altered *cryptochrome* (*cry^b^*;21D/TM6B) backgrounds were generated in Dr. Michael Rosbash' laboratory (HHMI, Brandeis Univ., USA) (Fig. 2). The UAS construct carrying line was UAS-mCD8-GFP, encoding a GFP-variant that is targeted to the cell membrane. The line was obtained from the Bloomington Stock Center.

To scrutinize the L2 morphology we used progeny of the following crosses of *D. melanogaster* line*s*: 21D-GAL4×UAS-mCD8-GFP; *per^01^*;21D/TM2×UAS-mCD8-GFP; *cry^b^*;21D/TM6B×UAS-mCD8-GFP.

All experiments were performed on adult flies, males and females, of *Drosophila melanogaster* transgenic lines mentioned above. Flies were raised on a standard cornmeal-yeast-sugar medium at 25°C in broad-spectrum light of 2,000 lx intensity during the light phase of the light/dark cycle (LD 12∶12 - 12 h of light and 12 h of darkness) and in continuous light (LL). During the dark phase of LD 12∶12 and in constant darkness (DD) experiments, flies were kept in a complete darkness. For experiments under DD or LL conditions, flies were held for 3 days in LD 12∶12 first for entrainment and next they were transferred to DD or LL.

Six days-old flies were decapitated under CO_2_ in a drop of fixative at four different time points in LD 12∶12: ZT1, ZT4, ZT13, ZT16 (ZT0 - the beginning of the day and ZT12 - beginning of the night) and in DD or LL: CT1, CT4, CT13, CT16 (CT0 – beginning of the subjective day and CT12 – beginning of the subjective night). For experiments conducted during the dark phase of LD 12∶12 and in DD, flies were decapitated under dim red light. A dissecting microscope illuminated with fiber-optic light guides equipped with red exit filters transmitting 590 nm wavelength light was used. The heads were fixed in 4% paraformaldehyde in 0.1 M PBS at 4°C for 4 h and cryoprotected overnight in 25% sucrose solution followed by embedding in OCT freezing medium (Sakura Finetek). To enhance GFP fluorescence in L2 cells, frozen sections (30 µm of thickness) were immunostained with the rabbit polyclonal anti-GFP primary serum, diluted 1: 1,000 (Nouvos Biological) followed by the goat anti-rabbit secondary antibody conjugated to Alexa Fluor 488, diluted 1: 1,000 (Invitrogen). The cryosections were mounted in Vectashield medium (Vector) and examined by a Zeiss Meta 510 Laser Scanning Microscope.

Morphological changes of the L2 dendrites were examined by tracing the outline of dendrites and axon of L2 cell cross sections at the same lamina depth, 15 µm from each L2 cell soma (Fig. 1C inset). Measurements were performed using ImageJ (v. 1.4 g with Java 1.6.0_05) and Zeiss LSM Image Examiner (v. 4.0.0.157) morphometric computer softwares.

In each experimental group (males or females collected at ZT1, ZT4, ZT13 and ZT16, or at CT1, CT4, CT13 and CT16) 9 to 15 flies were examined. In each individual fly 7–10 cells were measured and the mean values for each individual were calculated. The cross–sectional outlines of the L2 dendritic tree were calculated as the mean of means obtained from all individuals within a group. One–way analysis of variance (ANOVA) followed by Tukey's post hoc analysis was used to estimate statistically significant (*p*<0.05) differences between groups. Statistical analysis of the data was carried out using MS Excel and STATISTICA computer software.

## References

[1] Chen DM, Christianson JS, Sapp RJ, Stark WS (1992). Visual receptor cycle in normal and period mutant Drosophila: microspectrophotometry, electrophysiology, and ultrastructural morphometry.. Vis Neurosci.

[2] Pyza E, Meinertzhagen IA (1993). Daily and circadian rhythms of synaptic frequency in the first visual neuropile of the housefly's (*Musca domestica* L.) optic lobe.. Proc R Soc Lond B.

[3] Pyza E, Meinertzhagen IA (1995). Monopolar cell axons in the first optic neuropil of the housefly, *Musca domestica* L., undergo daily fluctuations in diameter that have a circadian basis.. J Neurosci.

[4] Pyza E, Meinertzhagen IA (1999). Daily rhythmic changes of cell size and shape in the first optic neuropil in *Drosophila melanogaster*.. J Neurobiol.

[5] Pyza E, Meinertzhagen IA (1997). Circadian rhythms in screening pigment and invaginating organelles in photoreceptor terminals of the housefly's first optic neuropil.. J Neurobiol.

[6] Cheng Y, Hardin PE (1998). Drosophila photoreceptors contain an autonomous circadian oscillator that can function without period mRNA cycling.. J Neurosci.

[7] Siwicki KK, Eastman C, Petersen G, Rosbash M, Hall JC (1988). Antibodies to the *period* gene product of *Drosophila* reveal diverse tissue distribution and rhythmic changes in the visual system.. Neuron.

[8] Hardin PE, Hall JC, Rosbash M (1990). Feedback of the *Drosophila period* gene product on circadian cycling of its messenger RNA levels.. Nature.

[9] Zerr DM, Hall JC, Rosbash M, Siwicki KK (1990). Circadian fluctuation of period protein mmunoreactivity in the CNS and the visual system of *Drosophila*.. J Neurosci.

[10] Ewer J, Frisch B, Hamblen-Coyle MJ, Rosbash M (1992). Expression of the *period* clock gene within different cell types in the brain of *Drosophila* adults and mosaic analysis of these cells' influence on circadian behavioral rhythms.. J Neurosci.

[11] Hardin PE (2005). The circadian timekeeping system of *Drosophila*.. Curr Biol.

[12] Helfrich-Förster C, Winter C, Hofbauer A, Hall JC, Stanewsky R (2001). The circadian clock of fruit flies is blind after elimination of all known photoreceptors.. Neuron.

[13] Ivanchenko M, Stanewsky R, Giebultowicz JM (2001). Circadian photoreception in *Drosophila*: function of cryptochrome in peripheral and central clocks.. J Biol Rhythms.

[14] Collins B, Mazzoni EO, Stanewsky R, Blau J (2006). *Drosophila* CRYPTOCHROME is a circadian transcriptional repressor.. Cell.

[15] Yu W, Zheng H, Houl JH, Dauwalder B, Hardin PE (2006). PER-dependent rhythms in CLK phosphorylation and E-box binding regulate circadian transcription.. Genes Dev.

[16] Kivimäe S, Saez L, Young MW (2008). Activating PER repressor through a DBT-directed phosphorylation switch.. PloS Biol.

[17] Martinek S, Inonog S, Manoukian AS, Young M (2001). A role for the segment polarity gene shaggy/GSK-3 in the *Drosophila* circadian clock.. Cell.

[18] Shafer OT, Rosbash M, Truman JW (2002). Sequential nuclear accumulation of the clock protein Period and Timeless in the pacemaker neurons of *Drosophila melanogaster*.. J Neurosci.

[19] Balys M, Pyza E (2001). Localization of the circadian clock controlling rhythms in the first neuropil of the housefly's optic lobe.. J Exp Biol.

[20] Pyza E, Cymborowski B (2001). Circadian rhythms in behaviour and in the visual system of the blowfly *Calliphora vicina*.. J Insect Physiol.

[21] Kula E, Pyza E (2007). Effects of locomotor stimulation and protein synthesis inhibition of circadian rhythms in size changes of L1 and L2 interneurons in the fly's visual system.. Dev Neurobiol.

[22] Górska-Andrzejak J, Keller A, Raabe T, Kilianek L, Pyza E (2005). Structural daily rhythms in GFP-labeled neurons in the visual system of *Drosophila melanogaster*.. Photochem Photobiol Sci.

[23] Pyza E, Górska-Andrzejak J (2004). Involvement of glial cells in rhythmic size changes in neurons of the housefly's visual system.. J Neurobiol.

[24] Brand AH, Perrimon N (1993). Targeted gene expression as a means of altering cell fates and generating dominant phenotypes.. Development.

[25] Krishnan B, Levine JD, Lynch MK, Dowse HB, Funes P (2001). A new role for *cryptochrome* in a *Drosophila* circadian oscillator.. Nature.

[26] Levine JD, Funes P, Dowse HB, Hall JC (2002). Advanced analysis of a cryptochrome mutation's effect on the robustness and phase of molecular cycles in isolated peripheral tissues of *Drosophila*.. BMC Neurosci.

[27] Cerani MF, Darlington TK, Staknis D, Mas P, Petti AA (1999). Light-dependent sequestration of timeless by *cryptochrome*.. Science.

[28] Busza A, Emery-Le M, Rosbash M, Emery P (2004). Roles of the two *Drosophila* cryptochrome structural domains in circadian photoreception.. Science.

[29] Miśkiewicz K, Schürmann F-W, Pyza E (2008). Circadian release of pigment-dispersing factor in the visual system of the housefly, *Musca domestica*.. J Comp Neurol.

[30] Górska-Andrzejak J, Salvaterra PM, Meinertzhagen IA, Krzeptowski W, Görlich A (2003). Cyclical expression of Na^+^/K^+^-ATPase in the visual system of *Drosophila melanogaster*.. J Insect Physiol.

[31] Mehnert Ki, Beramendi A, Elghazali F, Negro P, Kyriacou CP (2007). Circadian changes in *Drosophila* motor terminals.. Dev Neurobiol.

